# A “Rash” Decision in Anesthetic Management: Benzyl Alcohol Allergy in the Perioperative Period

**DOI:** 10.1155/2021/8859823

**Published:** 2021-06-24

**Authors:** Michael Tripp, Matthew Ribeiro, Susanna Kmiecik, Ramon Go

**Affiliations:** Department of Anesthesiology, Medstar Georgetown University Medical Center, Washington, DC, USA

## Abstract

Here, we present the case of a 54-year-old female presenting for outpatient ankle hardware removal who experienced severe total body pruritus along with a maculopapular rash persisting four days after the procedure. Patch testing demonstrated a sensitivity to benzyl alcohol, a preservative in propofol and several other anesthetics. The patient returned for left ankle arthroscopy a year later, and during that procedure, the anesthetic team avoided medications containing benzyl alcohol. This resulted in no pruritus or rash. Hypersensitivity reactions, ranging from contact dermatitis to anaphylaxis, are critical events in the perioperative period. Induction of general anesthesia has been implicated as the inciting event for perioperative hypersensitivity reactions. Benzyl alcohol is among a few excipients found in common anesthetic agents known to cause hypersensitivity reactions in susceptible patients. While reports of adult death are rare, infantile death due to benzyl alcohol has been described.

## 1. Introduction

Awareness and recognition of patient allergies are cornerstones of safe anesthesia administration. A recent meta-analysis demonstrated that 20% of the general population have contact allergies to common environmental allergens with broader data showing sensitization rates to one or more allergens among children ranging from 35 to 45% [1, 2]. While the data indicated that the reported incidence of perioperative anaphylaxis varies from 1 in 6,000 to 1 in 20,000 administrations of anesthesia, a recent report by Leif Saager et al. demonstrated that less-severe hypersensitivity reactions are significantly more common, with incidence rates as high as 1 in 677 patients in a tertiary-care academic medical center [3, 4]. They also postulated that even more subtle cases may have been missed clinically and thus underreported; the true incidence of hypersensitivity reactions is thus possibly even greater. Importantly, these mild cutaneous eruptions can herald more severe reactions upon reexposure to the offending agent. As a result, long-term patient safety outcomes can be bolstered through proper identification and analysis of possible patient hypersensitivities.

Adverse drug reactions will most likely occur during induction of anesthesia where numerous agents are administered to achieve intubating conditions, making the offending agent difficult to determine. Prompt recognition of a drug-induced hypersensitivity reaction is of paramount importance to deliver timely airway and cardiovascular support. Skin testing remains the gold standard in identifying the likely causative agent and may also help elucidate other cross-reactivities and sensitivities to provide alternative agents that would be safer for use in future cases [5]. More insidious than common allergy-inducing medications are pharmacologically inactive excipients in drug formulations. Although they are not the primary pharmaceutical agent, such additives can be biologically active. It has been long recognized that these can be “hidden hazards” for physicians using medications containing certain excipients; Holt et al. suggested that patients with documented allergies should use preservative-free anesthetics and consider intradermal testing [6, 7]. For example, early formulations of propofol with Cremophor EL as a biocompatible vehicle caused high incidences of anaphylaxis [8, 9]. Although newer lipid emulsions without this compound have been extremely efficacious and safe [3], there are still drawbacks, most notably for our case, excipients added for antimicrobial purposes, such as benzyl alcohol [10, 11].

## 2. Case Report

A 54-year-old woman with a history of hypertension, gastroesophageal reflux disease, obesity, and postoperative nausea and vomiting presented to our ambulatory surgery center for left ankle hardware removal from a previous history of left ankle fracture. Her only allergy was to contrast dye. She underwent a standard induction with propofol, midazolam, and fentanyl before a laryngeal mask airway was placed. Sevoflurane at 1 MAC was used for maintenance. The surgery was uneventful with successful emergence and LMA removal after one hour and thirty minutes of surgical time. Immediately in the postanesthesia care unit, the patient experienced severe total body pruritus along with a maculopapular rash. Her vital signs were stable, and the patient did not complain of any wheezing. She was given diphenhydramine in the recovery room and was then discharged to home. She reported that the rash improved over the course of four days with diphenhydramine. Patch testing performed by an allergy and immunology specialist showed sensitivity to benzyl alcohol, a propofol preservative.

The patient presented again seven months later for left ankle arthroscopy and posterior tibial tendon debridement. The patient underwent general endotracheal anesthesia with etomidate, midazolam, fentanyl, and succinylcholine for induction and intubation. Sevoflurane was used for anesthetic maintenance. The surgical procedure was uneventful, and the patient was successfully extubated. The patient reported a significant difference in her immediate postoperative period with minimal to no pruritus. A phone call was made to the patient three weeks later, and the patient denied any pruritus or rash compared to her previous postsurgical course. The patient expressed gratitude for avoiding the pruritus and rash. The patient provided consent for this case report.

## 3. Discussion and Conclusion

Benzyl alcohol is oxidized aromatic alcohol used as an excipient or additive to medications, and while it is considered pharmacologically inactive, it is not a benign substance. Benzyl alcohol is used in many products primarily as a bactericidal agent by direct destruction of the bacterial cell membrane upon contact [12]. Given its use in many products, there have been documented contact dermatitis reactions in topical and injectable solutions [5]. Contact dermatitis produces a type IV hypersensitivity reaction, also known as delayed or cell-mediated hypersensitivity reaction. This reaction is a dose-dependent T-cell-mediated response [7, 13]. Type IV hypersensitivity reactions occur when a hapten or other nonimmunogenic substances combine with a carrier protein to become immunogenic. The hapten is then recognized by hapten-specific T cells in lymph nodes and undergoes sensitization (see Table 1). On subsequent exposures, T cells will undergo an elicitation phase, or become activated, and quickly be recruited to the skin to cause inflammation.

Signs and symptoms of delayed hypersensitivity reactions are not as severe as anaphylaxis, a type I hypersensitivity reaction or an immediate hypersensitivity reaction (see Table 1). In type I hypersensitivity reactions, an allergen interacts with IgE on mast cells and basophils that immediately releases preformed mediators, cytokines, and enzymes that act locally and recruit more inflammatory cells [7, 13]. Benzyl alcohol's allergic potential has been known for some time as evidenced by documented type IV hypersensitivity reactions; however, cases of severe allergic reaction and toxic effects do exist, although rare [5].

The most well-documented toxic effect of benzyl alcohol in products comes from a case series published by Gershanik et al. [14]. They described ten cases of premature infants receiving medications containing benzyl alcohol and developing a series of manifestations termed the “gasping syndrome.” The infants received multiple injections of heparinized bacteriostatic sodium chloride for flushing indwelling catheters as well as various medications (antibiotics, sodium bicarbonate, and calcium) reconstituted in bacteriostatic water containing 0.9% benzyl alcohol. Symptoms of gasping syndrome include neurological deterioration, gasping respirations, intracranial hemorrhage, severe metabolic acidosis, hematologic abnormalities, skin breakdown, hepatic and renal failure, and cardiovascular collapse. All ten infants died and, on analysis of blood and urine, had 50 times the safe adult dosing of benzyl alcohol. Benzyl alcohol is eliminated through conjugation in the liver with glycine and excreted by the urine as hippuric acid. Premature infants have impaired metabolism and elimination of benzyl alcohol causing dangerous accumulation. Benzyl alcohol has more recently been removed from medications commonly administered to infants [14], but there is still the possibility of infants receiving benzyl alcohol-containing medications if nonroutine medications are given, such as clindamycin. Being aware of this possibility is imperative in settings where nonroutine medications are given to infants more often, such as in the intensive care units.

Hypersensitivity reactions in adults are not as profound as infantile toxic reactions and are rarely seen systemically. In our case, we were able to see a previous reaction to anesthetic agents and, on patch testing, confirm that the causal agent was benzyl alcohol. Benzyl alcohol is utilized in many drugs administered intraoperatively, specifically propofol, midazolam, and dexamethasone in our case. Other medications containing benzyl alcohol are listed in Table 2. Knowing which medications may cause a reaction in our patient, we were able to adjust the standard induction to include etomidate rather than propofol, a main contributor of her hypersensitivity reaction, with a satisfactory outcome. Of note, we did not discover midazolam as a benzyl alcohol-containing medication until creating this case report, so it was administered to our patient. Our patient did not have a hypersensitivity reaction, perhaps because of the small volume used, but previous exposure to benzyl alcohol has led to death in at least one documented patient who received diazepam infusion [15].

Avoiding benzyl alcohol-containing anesthetics can be difficult. Abstaining from propofol can pose a challenge in many cases for its variety of uses in sedation, induction and maintenance of anesthesia, rapid deepening of anesthetic levels, and blood pressure control. Due to potential adverse effects of benzyl alcohol allergy, the authors suggest that anesthesia providers should consider peripheral nerve blocks, neuraxial anesthesia, or avoiding benzyl alcohol-containing agents when applicable (see Table 2). The authors also suggest that patients with postanesthetic signs or symptoms of severe allergic reaction should consider patch testing to help guide future anesthetic management. Given the possibility of anaphylaxis and death in an understudied excipient, it is imperative to know which products contain benzyl alcohol and in which patients to avoid their use.

## Figures and Tables

**Table 1:** Gell and Coombs classification of hypersensitivity reactions distinguishes four types of immune responses [7, 13].

Type	Description	Mechanism	Clinical features
I	IgE-mediated, immediate-type hypersensitivity	Antigen exposure causes IgE-mediated activation of mast cells, causing the release of vasoactive substances (e.g., histamine) and inflammatory mediators)	Anaphylaxis Angioedema Bronchospasm Urticaria

II	Antibody-dependent cytotoxicity	Antigen/haptens on cells bind IgG and/or IgM antibodies, leading to cell or tissue injury via the complement (formation of the membrane attack complex) or phagocytosis	Hemolytic anemia Thrombocytopenia Neutropenia

III	Immune complex disease	Deposition of antigen-antibody complexes in vessels or tissue, leading to complement activation and recruitment of neutrophils by the interaction of immune complexes with Fc IgG receptors	Serum sickness Arthus reaction Glomerulonephritis

IV	Cell-mediated delayed-type hypersensitivity	Sensitized T lymphocytes encounter the antigen via MHC presentation, causing activation, which then mediates tissue injury	Contact dermatitis Interstitial nephritis Drug-induced hepatitis Transplant rejection PPD skin test

**Table 2:** Pharmacological agents containing benzyl alcohol often used in anesthesia.

Amiodarone	Glycopyrrolate	Midazolam
Clindamycin	Heparin	Methylprednisolone
Dexamethasone	Hydrocortisone	Morphine
Diazepam	Lorazepam	Propofol
